# Pancreas Transplantation: Does Age Increase Morbidity?

**DOI:** 10.1155/2011/596801

**Published:** 2011-06-04

**Authors:** Cheguevara Afaneh, Barrie S. Rich, Meredith J. Aull, Choli Hartono, David B. Leeser, Sandip Kapur

**Affiliations:** ^1^Division of Transplantation, Department of Surgery, New York Presbyterian Hospital, Weill Cornell Medical College, 525 East 68th Street, P.O. Box 207, New York, NY 10021, USA; ^2^Division of Nephrology, Department of Medicine, Weill Cornell Medical College, New York, NY 10021, USA

## Abstract

*Introduction*. Pancreas transplantation (PTx) is the only definitive intervention for type 1 diabetes. Medical advancements in diabetes care have led to an aging PTx candidate pool. We report our experience with patients ≥50 years of age undergoing PTx. *Methods*. We reviewed 136 consecutive PTx patients at our institution from 1996–2010; 17 were ≥50 years of age. We evaluated demographics, surgical complications, acute rejection (AR) rates, nonsurgical infections, and survival outcomes. *Results*. Demographic data was similar (*P* > .05) between groups, excluding age. The two groups had comparable major and minor surgical complication rates (*P* = .10 and *P* = .25, resp.). The older group had a lower 1-year and overall AR rate (*P* = .04 and *P* = .03, resp.). The incidence of non-surgical infections and overall patient and graft survival was similar between groups (*P* > .05). *Conclusion*. Older patients with type 1 diabetes are feasible candidates for PTx, as surgical morbidity, incidence of infections, and AR rates are low.

## 1. Introduction

Pancreas transplantation remains the best intervention for type 1 diabetes mellitus that reestablishes normoglycemia without the need for insulin therapy. Improvements in the physician's armamentarium to reduce the development of diabetic complications have lead to the emergence of a healthier aging population of type 1 diabetics in need of pancreas transplantation [1, 2]. Progress in surgical techniques, critical care, and immunosuppressive medications have also expanded the pool of transplant candidates to include a significant proportion of patients over the age of 50 [3].

Despite continued improvements in the field of transplantation, pancreas transplantation has the highest morbidity of all routinely performed abdominal solid organ transplantation procedures [2, 4]. To further complicate this procedure, the transplant pool often consists of older diabetic patients, who will naturally have more preoperative comorbidities than their younger counterparts. Thus, weighing the risk of the pancreas transplantation procedure against the benefit of insulin independence has been debatable [5, 6]. Salvalaggio et al. demonstrated a lower mortality in patients receiving pancreas transplants, regardless of age, than those remaining on the waiting list, even with older (45 years or older) donors, compared to remaining on the waiting list [7].

Many institutions have placed age limitations on potential pancreas transplant recipients, considering age a risk factor for inferior outcomes. In one study patients 50 years of age or older had a higher incidence of graft thromboses and bleeding requiring re-exploration, as well as a higher incidence of pulmonary infections [6]. Other studies have shown a lower patient survival for older patients (45 years or older) undergoing pancreas transplantation [8, 9]. Additionally, Gruessner and Sutherland found that patients older than 45 years of age had lower graft survival; however, immunological graft loss decreased with increasing age [9]. 

The mean age of pancreas transplant recipients continues to increase; however, the data on older pancreas transplant recipients remains limited. The intent of our retrospective study was not only to demonstrate the feasibility of pancreas transplantation in older recipients, but also to assess surgical morbidity, infectious risks, and overall outcomes of this patient population.

## 2. Methods

From October 1996 to July 2010, 136 pancreas transplants were performed in 131 patients. Of these 136 transplants, 92 were simultaneous pancreas-kidney (SPK) transplants, 37 were pancreas after kidney (PAK) transplants, and 7 were pancreas transplant alone (PTA). One hundred twelve pancreas transplants were enterically drained, while the remaining 24 had bladder drainage. All pancreas transplants had systemic venous drainage. All patients were type 1 diabetics on insulin therapy preoperatively (C-peptide negative). We separated the recipients into two groups based on age: those younger than 50 years of age and those 50 years of age or greater. 

Recipient baseline data reviewed included age, gender, body mass index, age of diagnosis of diabetes, duration of diabetes, preoperative hemodialysis requirement, comorbidities, serum albumin within 30 days of transplantation, incidence of Hepatitis C virus infection (HCV), and cytomegalovirus (CMV) status. Donor and perioperative data included donor age, donor CMV status, induction therapy, cold ischemia time, and number of human leukocyte antigen (HLA) mismatches.

Perioperative and postoperative parameters included total operative time, estimated blood loss, length of hospital stay, complications in the first 30 days including graft thrombosis, acute rejection rate, and incidence of delayed graft function (defined as the need for hemodialysis within the first week after transplant). Hemoglobin A1c (HgA1c) levels were analyzed at 6 months, 1, 1.5, 2, and 3 years posttransplant. Acute rejection episodes were defined by biopsy-proven results or empiric treatment with corticosteroids. Complications were graded using the modified Clavien system, which categorizes surgical complications based on the degree of intervention needed [10]. Minor complications were classified as grade I-II, while major complications were classified as grade III-V. Postsurgical infectious complications included superficial surgical site infections, urinary tract infections, abscesses, and fever of unknown origin. Gastrointestinal complications included small bowel obstructions and ileus, while the urinary complication was hematuria treated with urinary catheterization. Hematological complications included acute blood loss anemia and vessel thrombosis treated with anticoagulation. The cardiovascular complications included postoperative arrhythmias and acute myocardial infarction. Re-exploration was defined as any operative procedure involving the intraperitoneal or retroperitoneal space. Indications for re-operation included open abdominal washouts, graft thrombosis, hernias/evisceration, major hemorrhage, anastomotic site leak, and lysis of adhesions. All non-surgical infectious complications were classified as bacterial, viral, or fungal. Incidence of Posttransplant Lymphoproliferative Disease (PTLD) was also recorded.

All potential transplant recipients were evaluated by a multidisciplinary team. All potential recipients, irrespective of age, undergo a rigorous preoperative work-up, including cardiology clearance, a nuclear stress test, and an echocardiogram. All patients with unrevascularized coronary artery disease underwent revascularization procedures prior to transplantation. Additionally, older recipients undergo colonoscopy (age >50 years), mammography (women age >40 years), and prostate-specific antigen testing (men age >50 years) during the preoperative work-up. The following criteria are considered contraindications to pancreas transplantation at our institution: active infection, active malignancy, active liver disease, unrevascularized coronary artery disease, a history of noncompliance, active substance abuse, or patients with significant psychiatric disorders. 

At our institution, induction therapy was not routinely administered during pancreas transplantation until 2001. Most patients received an IL-2 Receptor Antibody (IL-2RA), while a small minority received rabbit Antithymocyte Globulin (ATG). The immunosuppression regimen consisted of a calcineurin inhibitor (Tacrolimus or Cyclosporin), mycophenolate mofetil, and low-dose prednisone. Immunosuppression regimens and doses were similar for both younger and older recipients. Calcineurin inhibitors were dosed based on the same target trough levels for both groups. All patients received prophylaxis with bactrim for 1 year and valganciclovir for 6 months (3 months for donor negative/recipient negative). All patients were routinely placed on Aspirin therapy postoperatively. 

Statistical analyses were performed using Graphpad Prism software version 5.03 (GraphPad Software, Inc. La Jolla, CA). All data are listed as mean ± standard deviation (SD), unless otherwise specified. Categorical variables were compared used chi-square test or Fisher's exact test, while continuous variables were compared using Student's *t*-test (two-tailed). Patient and death-censored graft survival rates were calculated using the Kaplan-Meier method including the log-rank test. A *P-*value less than  .05 was considered statistically significant.

## 3. Results

Between October 1996 and July 2010, 131 patients underwent 136 consecutive pancreas transplants at our institution. Seventeen pancreas transplants (13%) were performed in patients ≥50 years of age, while the remaining 119 pancreas transplants (87%) were performed in patients <50 years of age. The ≥50 years of age group consisted of 11 SPKs and 6 PAKs with a median age of 53 (range 50–61), while the <50 years of age groups consisted of 81 SPKs, 31 PAKs, and 7 PTAs with a median age of 37 (range 19–49). 

Patient demographics and donor parameters are listed in Table 1. As expected, the mean [±SD] recipient age was significantly higher in the ≥50 years of age group (54 ± 3.2 versus 37 ± 7.0,  *P* < .0001). However, there was no difference between the two groups with respect to proportion of males, body mass index, preoperative serum albumin, proportion of patients on hemodialysis preoperatively, or proportion of patients infected with HCV (*P* > .05). Older recipients developed type 1 diabetes at an older age (21 ± 9.2 years of age) compared to younger recipients (13 ± 6.5 years, *P* = .001). In addition older patients had a longer duration of diabetes (33 ± 9.0 years versus 24 ± 6.8 years, resp.; *P* = .0004). Additionally, older recipients were more likely to have a history of peripheral vascular disease (53% versus 27%, *P* = .03) and/or coronary artery disease (47% versus 18%, *P* = .006), but not hypertension (*P* = .85) or cerebrovascular disease (*P* = .12). The proportion of enterically drained pancreata, induction therapy, donor age, cold ischemia time, and number of HLA mismatches was similar between the two groups (Table 1). The mean [±SD] follow-up time for the two groups was similar (5.9 ± 4.5 years versus 4.8 ± 3.7 years, *P* = .48). 

**Figure 1:**
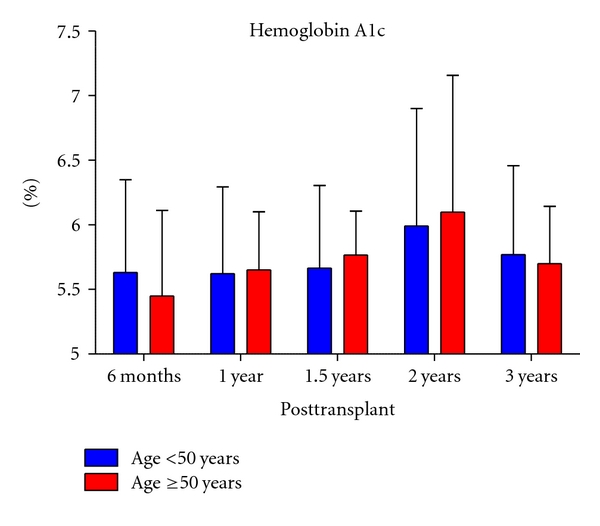
Pancreas allograft function. The bar graph illustrates the mean hemoglobin A1c (HgA1c) values for younger recipients (blue) and older recipients (red). The error bars represent the standard deviation. The *Y*-axis is the percent glycosylated hemoglobin present and the *X*-axis is the number of years posttransplant for each group. There was no significant difference in HgA1c at 6 months, 1, 1.5, 2, and 3 years after transplant between the groups (*P* > .05).

Table 2 lists the perioperative and postoperative parameters. The total operative time (*P* = .70) and length of hospital stay was similar between the two groups (*P* = 1.0). The older patients trended towards more blood loss (*P* = .06). There was no difference between the groups with respect to incidence of graft thrombosis (*P* = 1.0) and percentage of patients experiencing a complication (*P* = .85) between the two groups. The one year and overall acute rejection rates, defined by the number of patients experiencing at least one episode of acute rejection in the given timeframe, were significantly lower in the ≥50 years of age group (5.9% versus 30.2%, *P* = .04 and 11.8% versus 38.7%, *P* = .03, resp.). The incidence of delayed graft function was similar between the two groups (*P* = .54). Finally, there was no difference in mean HgA1c values at 6 months, 1, 1.5, 2, and 3 years after transplant (*P* > .05) (Figure 1).

The distribution of complications is listed in Table 3 for both the <50 years of age group and the ≥50 years of age group. There was no difference between the two groups in all categories (*P* > .05) except hematological complications (*P* = .02). The rates of minor and major complications per patient were not different between the two groups (*P* = 10 and *P* = .25, resp.).

Non-surgical infectious complications were categorized based on infectious etiology in Table 4. The average number of bacterial, viral, and fungal infections per patient in each group was not significantly different (*P* > .05). Furthermore, the number of infections requiring hospitalizations was not different between the two groups (*P* > .05). Finally, the incidence of CMV infections and PTLD was also similar between the two groups (*P* > .05).

**Figure 2:**
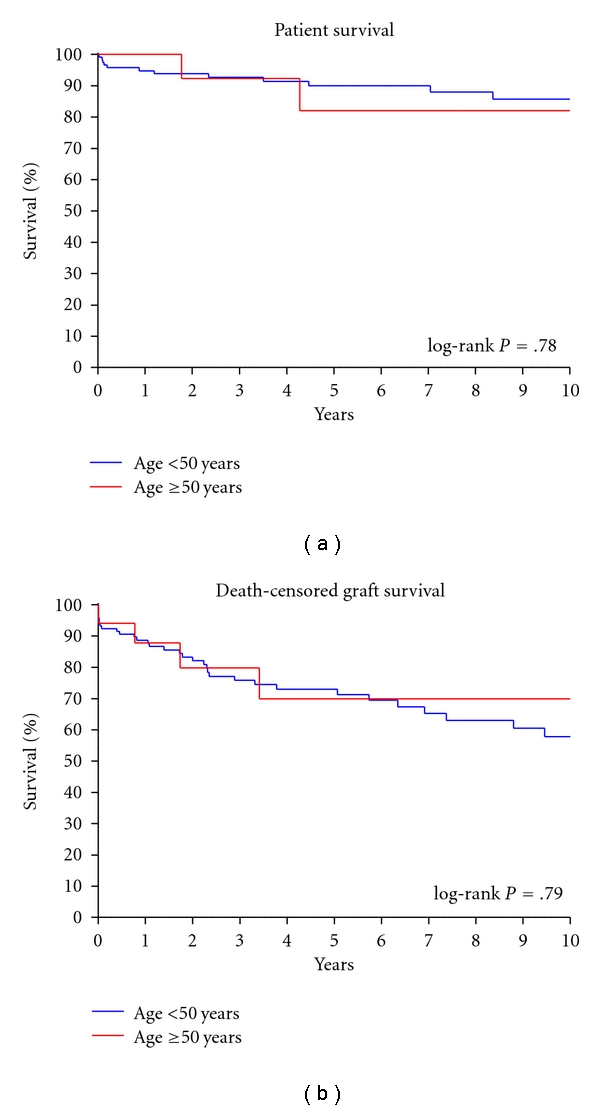
Kaplan-Meier survival curves. The overall patient survival is depicted in (a), while the death-censored graft survival is depicted in (b). Survival curves were calculated using the Kaplan-Meier method. Patient and death-censored graft survival rates were similar between the two groups (log-rank *P* = .78  & log-rank *P* = .79, resp.). The median follow-up time was 5.1 years in the <50 years of age group and 4.3 years in the ≥50 years of age group.

The overall patient survival was similar between the two groups (Figure 2(a), log-rank *P* = .79). The 1-year patient survival rates for the younger and older groups were 95% and 100%, respectively. The 3- and 5-year patient survival in the <50 years of age group was 93% and 90%, and 92% and 82% in the ≥50 years of age group. The overall death-censored graft survival was similar between the two groups (Figure 2(b), log-rank *P* = .79). The 1 year death-censored graft survival for the <50 years of age and ≥50 years of age groups was 89% and 88%, respectively. The 3- and 5-year death-censored graft survival rates in the <50 years of age group was 76% and 73%, and 80% and 70% in the ≥50 years of age group.

## 4. Discussion

Pancreas transplantation remains the most effective method to achieve prolonged periods of normoglycemia. Our study demonstrates that type 1 diabetics 50 years of age or older can successfully undergo pancreas transplantation with favorable outcomes. Additionally, these patients demonstrated a lower acute rejection rate than younger patients, without an increased risk of infectious complications. Moreover, surgical morbidity was not increased in this older patient population. 

The pool of pancreas transplant recipients has steadily aged over the past decade [11]. As novel therapies for type 1 diabetes continue to improve patient outcomes and reduce complications, pancreas transplant candidates will continue to increase in age and therefore have increasing comorbidities. This, in combination with a high perioperative complication rate (including re-exploration), compared to other solid organ transplantation procedures, calls for critical evaluation of suitable candidates for pancreas transplantation [4, 12]. In Europe, the age limit for pancreas transplantation candidates was originally set at 45 years [13]. Over more than a decade, the maximum age limit has increased, although no formal consensus exists. Several studies demonstrated increased morbidity and mortality for older pancreas transplant recipients and even advocated for only transplanting younger patients [14, 15]. In one study, a survival advantage for pancreas transplant candidates with end-stage renal disease was seen in every age category, except when patients were 50 years of age or older [16]. Nevertheless, more recent studies have demonstrated similar outcomes for older and younger patients with respect to graft and patient survival [2, 6].

In our current study the number of patients experiencing a major or minor complication in the two age groups was not significantly different, despite increased comorbidities in the older group. Hematologic complications were the most frequent type of complication encountered in the 50 years of age or older group and this was the only type of complication in which a significant difference was seen between the younger and older cohorts. This may be related to the trend towards increased intraoperative blood loss in the patients 50 years of age or greater. The most common complication experienced in the less than 50 years of age group were those requiring re-exploration. These results are in contrast to Ablorsu et al. who found that patients 50 years of age or older had a higher incidence of bleeding requiring re-exploration [6]. In another study by Freise et al. older recipients (age 49 years or older) had similar technical complication rates as recipients less than 49 years of age [14]. Similarly, Schenker et al. showed no difference in complications requiring repeated laparotomy between older and younger recipients [2]. 

A prominent cause of early graft loss cited in the literature in pancreas transplantation has been vascular thrombosis of the allograft [8]. In our study we found that the ≥50 years of age group did not have an increased incidence of graft thrombosis. Ablorsu et al. demonstrated similar survival between older and younger patients; however, illustrated that patients aged 50 years or older had a higher rate of graft thrombosis compared to patients younger than 50 years of age [6]. In another study by Schenker and colleagues, the venous graft thrombosis rates were similar between older (14%) and younger recipients (11%) [2]. 

Immunological graft loss is an established cause of graft failure. Our study shows a significantly lower one-year acute rejection rate in patients aged 50 years or older. This could be explained by the depressed immunological response associated with senescence [17]. Tesi et al. demonstrated in a large series of kidney transplant recipients a lower rate of acute rejection in patients 60 years of age or older, citing senescence as a likely contributor [18]. Senescence in studies such as this typically refers to patients aged 60 and older. Nevertheless, similar to our study Gruessner and Sutherland noticed a lower acute rejection rate in patients aged 45 years or older, compared to younger patients [9]. Several other studies, in contrast, did not see a significant difference in the acute rejection rates between older and younger recipients [2, 6]. It is noteworthy that in this study, induction therapy was used in almost 80% of older recipient transplants, while almost 50% of the cases in the younger group had no induction therapy administered.

In this study we noted similar postoperative infectious complications between recipients less than 50 years of age and those 50 years of age or older. Furthermore, the overall infection rate over the lifespan of the allograft was similar between the two groups, regarding bacterial, viral, and fungal infections. It is well known that elderly patients have a higher incidence of pulmonary infections following surgery. Some contributors to this increased risk include de-conditioning of respiratory musculature, poor nutritional status, and impaired immune response associated with senescence [17, 19]. In one study, older patients experienced an increased incidence of chest and pulmonary infections following pancreas transplantation [6]. We, however, did not see an increased risk of any type of infection in this older population, including CMV infection. The incidence of PTLD was over 7 times greater in the older age group than the younger group; however, this was not found to be statistically significant. This is likely due to the small sample size of older patients, and these patients should be evaluated regularly for the development of PTLD. 

Patients 50 years of age or older experienced similar short- and long-term graft and patient survival as patients less than 50 years of age. The 1, 3, and 5 year patient survival rates were 100%, 92%, and 82% in the patients aged ≥50 years of age and 95%, 93%, and 90% in patients younger than 50 years of age, respectively. The death-censored graft survival rates at 1, 3, and 5 years were 88%, 80%, and 70% in the older group and 89%, 76%, and 73% in the younger group. The 1-year patient and graft survival rates in the 50 years of age or older group is slightly better than similar sized study (graft survival 88% versus 74% and patient survival 100% versus 92%)[6]. On a larger scaled metric, our graft and patient survival rates are comparable to pancreas transplant recipients in the United States and abroad [20, 21].

This study is not without limitations. This is a retrospective analysis from a single institution with a small sample size and with the clear limitations of chart review. However, our proportion of patients aged 50 or older (13%) is similar to that found elsewhere in the United States (12%–15%) [9]. From 1996 to 2000, our recipients aged 50 years or older compromised 7% of all pancreas transplant recipients. In contrast, from 2001 to 2010, older recipients represented almost 17% of all pancreas transplants at our institution. Finally, there may be a bias in the follow-up schedule of older patients. Clinicians may follow these patients more stringent and frequent than younger patients, which may contribute to the favorable outcomes.

This study demonstrates comparable outcomes of older and younger recipients of pancreas allografts, and encouraging outcomes of our older patients compared to previously published literature reports. This is likely multifactorial in nature. The evolution of more sophisticated immunosuppressive medications, coupled with innovation in surgical techniques and advances in critical care may all contribute to our results. Additionally, our older patient population is carefully screened by medical, surgical, and cardiac consultants prior to transplantation. All patients undergo revascularization procedures before their consideration as potential recipients. Preoperatively, our older patients demonstrated excellent nutritional status, as evidenced by their preoperative serum albumin values. Meticulous donor selection is an equally important contributor to our favorable older recipient outcomes, including young donors (mean age 32 years) and short cold ischemia times (<8 hours). Finally, older recipients may have been followed more rigorously and possibly more frequently than younger patients, which may contribute to the lower acute rejection rate.

## 5. Conclusions

In summary our data suggests that patients 50 years of age or older are suitable candidates for pancreas transplantation with excellent short- and long-term outcomes. In our experience older patients did not experience increased surgical morbidity, infectious complications, or inferior patient and graft survival rates compared to younger patients. These patients may experience a lower acute rejection rate. With careful patient selection and thorough medical assessment, older patients should be considered potential candidates to receive a pancreas allograft.

##  Author Contributions and Conflicts of Interest

C. Afaneh: Participated in research design, writing of the paper, in the performance of the research, and in data analysis, no conflict of interest, B. Rich: Participated in research design, writing of the paper, and in the performance of the research, no conflict of interest, M. Aull: Participated in research design, and drafting article, no conflict of interest, C. Hartono: Participated in research design, no conflict of interest, D. Lesser: Participated in research design, and drafting article, no conflict of interest, S. Kapur: Participated in research design, no conflict of interest.

## Figures and Tables

**Table 1:** Patient characteristics and donor parameters.

	<50 years of age *N* = 119	≥50 years of age *N* = 17	*P* value
Male, *n *(%)	60 (50%)	11 (65%)	.27
Recipient Age	37 ± 7.0	54 ± 3.2	<.0001
Body Mass Index (kg/m^2^)	25.0 ± 5.0	26.1 ± 4.0	.30
Age at diagnosis of type 1 DM (years)	13 ± 6.5	21 ± 9.2	.001
Duration of type 1 DM prior to transplant (years)	24 ± 6.8	33 ± 9.0	.0004
Preoperative Hemodialysis, *n* (%)	99 (83%)	13 (76%)	.50
HCV, *n* (%)	5 (4%)	0 (0%)	1.0

Comorbidities:			
Hypertension, *n* (%)	60 (50%)	9 (53%)	.85
Peripheral Vascular Disease, *n* (%)	32 (27%)	9 (53%)	.03
Coronary Artery Disease, *n* (%)	21 (18%)	8 (47%)	.006
Cerebrovascular Disease, *n* (%)	3 (3%)	2 (12%)	.12

Preoperative serum albumin (g/dl)	3.80 ± 0.58	3.86 ± 0.64	.98

CMV Status:			
D+/R+, *n* (%)	39 (33%)	4 (24%)	
D−/R+, *n* (%)	19 (16%)	3 (18%)	.90
D+/R−, *n* (%)	31 (26%)	5 (29%)	
D−/R−, *n* (%)	30 (25%)	5 (29%)	

SPK, *n* (%)	81 (68%)	11 (65%)	
PAK, *n* (%)	31 (26%)	6 (35%)	.47
PTA, *n* (%)	7 (6%)	0 (0%)	

Enteric Drainage, *n* (%)	100 (84%)	16 (94%)	.47
Induction therapy used (yes), *n* (%)	65 (55%)	13 (77%)	.12

Induction Agent:			
None, *n* (%)	54 (45%)	4 (23%)	
IL-2RA, *n* (%)	51 (43%)	10 (59%)	.23
Antithymocyte Globulin, *n* (%)	14 (12%)	3 (18%)	

Donor Age (years)	30 ± 10.4	32 ± 10.7	.52
Pancreas Cold Ischemia Time (hrs)	7.8 ± 3.1	7.2 ± 3.8	.52
HLA Mismatches	4 ± 1.2	4 ± 1.8	.41
Duration of follow-up (years)	5.9 ± 4.5	4.8 ± 3.7	.48

DM = Diabetes mellitus, D = Donor, R = Recipient, IL-2RA = Interleukin-2 Receptor Antagonist, HLA = Human Leukocyte Antigen.

**Table 2:** Perioperative and postoperative parameters.

	<50 years of age *N* = 119	≥50 years of age *N* = 17	*P* value
Total operative time (min)	283 ± 92.9	271 ± 73.3	.70
Estimated blood loss (ml)	571 ± 510.1	730 ± 399.2	.06
Length of Hospital Stay (days)	12 ± 9.1	11 ± 8.0	.61
Graft Thrombosis, *n* (%)	13 (11%)	2 (12%)	1.0
No. of Patients Experiencing a Complication, *n* (%)	59 (50%)	8 (47%)	.85
Delayed Graft Function, *n* (%)	5/81 (6%)	1/11 (9%)	.54
One Year Acute Rejection Rate	30.2%	5.9%	.04
Overall Acute Rejection Rate	38.7%	11.8%	.03

**Table 3:** Distribution of surgical complications.

Classification	<50 years of age *N* = 66^a^	≥50 years of age *N* = 10^a^	*P* value
Infectious, *n* (%)	14 (21%)	1 (10%)	.68
Gastrointestinal, *n* (%)	4 (6%)	1 (10%)	.52
Urinary, *n* (%)	1 (1%)	0 (0%)	1.0
Hematological, *n* (%)	6 (9%)	4 (40%)	.02
Cardiovascular, *n* (%)	3 (5%)	1 (10%)	.44
Radiological Drainage of a Collection, *n* (%)	9 (14%)	1 (10%)	1.0
Re-operation, *n* (%)	29 (44%)	2 (20%)	.19
Minor complications per patient (mean)	0.14	0.35	.10
Major complications per patient (mean)	0.41	0.24	.25

^a^refers to number of complications in each group.

**Table 4:** Non-surgical infectious complications.

	<50 years of age *N* = 119	≥50 years of age *N* = 17	*P* value
Avg. No. of Bacterial Infections/Patient	2.25	2.47	.72
Avg. No. of Viral Infections/Patient	0.39	0.35	.88
Avg. No. of Fungal Infections/Patient	0.13	0.06	.27
Avg. No. of Infections Requiring Hospitalization/Patient	0.86	1.18	.53
Incidence of CMV, *n* (%)	22 (18%)	3 (18%)	.93
PTLD, *n* (%)	1 (0.8%)	1 (5.9%)	.24

CMV: Cytomegalovirus, PTLD: Posttransplant lymphoproliferative disorder.
